# Stretching the Optic Nerve

**Published:** 1882-04

**Authors:** 


### STRETCHING THE OPTIC NERVE.

Dr. Herman Kümmel, of Hamburg (*Deutsche Med. Wochenschr.*, No. 1, 1882), relates several cases of optic-nerve stretching. The operation is performed as follows : The patient is anæsthetized and the lids separated by means of a speculum. The conjunctiva is then divided for a distance of nearly half an inch, at the outer corneal border. The conjunctiva is dissected up to a sufficient depth and a strong strabismus hook is carried back between the inferior and external recti muscles. The hook is made to hug the eyeball closely until the optic nerve is caught, which is then strongly stretched several times. The optic nerve is felt as a tense, firm band. The sensation conveyed to the hand by the nerve must be acquired by experiments on the cadaver. The eyeball can be steadied by forceps. The operation was done by Kümmel seven times on three cases.

The first case was a girl, nine years of age, who had been totally blind for two years. The left optic was stretched twice on August 8th, 1881, and the operation repeated on the same nerve at the end of eight days. Six days after, the right optic was also stretched. No bad results whatever followed the operation, but no improvement or other appreciable change took place in the eye.

The second case, a man of 44, had atrophy of the optic nerves, brilliant white discoloration of the papillæ. Color-sense entirely gone. History of syphilis fifteen years ago. Counts fingers at two feet distance with right eye, not at all with left eye. Veins and arteries both contracted. Two months after the operation, counts fingers at two feet with left eye and at six feet with right eye. The left optic nerve had been twice stretched. A remarkable fact was that the improvement in the right eye—not operated on—was greater than in the left.

The third case, a man of 45, had been affected with tabes dorsalis for three years. Vision in the left eye was entirely absent, while with the right eye fingers could be counted at one and a half feet distance. Both optic nerves were stretched at one sitting. No bad results from the operation except swelling of the conjunctiva, which disappeared under appropriate treatment in the course of a week. No improvement in vision after the operation.

The effects of the operation may be summarized as follows : There is a change in the circulation in the retina manifested by an increase of blood in the veins and perhaps also in the arteries. The ophthalmoscope appears to show a transitory œdema. No hemorrhages into the retina or intraocular end of the optic nerve have been observed ; neither has inflammation of the nerve occurred, in spite of the somewhat severe lesion to the nerve tissues from the operation.

The indications for the operation are slowly progressive atrophy of the optic nerve, not accompanied by inflammatory complications. It seems especially indicated in syphilitic and tabetic processes, particularly if the amaurosis is not too advanced.

Possibly, intracranial affections of the centre of vision might be favorably influenced by stretching the optic nerve. A thorough opthalmoscopic examination must be made in all cases, in order to determine with exactitude whether the conditions present justify or demand the operation.

